# Folic acid modulates the Notch1/NF-κB pathway in a rat model of lipopolysaccharide-activated hippocampal microglia

**DOI:** 10.3389/fnana.2026.1748518

**Published:** 2026-04-10

**Authors:** Marwa Abd El-kader, Eman A. E. Farrag, Randa El-Gamal, Eman Mohamed El Nashar, Areej M. Alshehri, Rashid A. Aldahhan, Khulood M. Al-khater, Sara El-Desouky, M. W. El-Sherbeni, Neven A. Ebrahim

**Affiliations:** 1 Department of Human Anatomy and Embryology, Faculty of Medicine, Mansoura University, Mansoura, Egypt; 2 Department of Anatomy, Faculty of Medicine, Al-Baha University, Al-Baha, Saudi Arabia; 3 Department of Clinical Pharmacology, Faculty of Medicine, Mansoura University, Mansoura, Egypt; 4 Department of Medical Biochemistry and Molecular Biology, Faculty of Medicine, Mansoura University, Mansoura, Egypt; 5 Medical Experimental Research Center (MERC), Faculty of Medicine, Mansoura University, Mansoura, Egypt; 6 Department of Medical Biochemistry, Horus University in Egypt (HUE), New Damietta, Egypt; 7 Department of Anatomy, College of Medicine, King Khalid University, Abha, Saudi Arabia; 8 Clinical Anatomy Department, Faculty of Medicine, King Abdulaziz University, Jeddah, Saudi Arabia; 9 Department of Anatomy, College of Medicine, Imam Abdulrahman Bin Faisal University, Dammam, Saudi Arabia; 10 Medical Experimental Research Center, Faculty of Medicine, Mansoura University, Mansoura, Egypt; 11 Department of Chemistry, Faculty of Science, Mansoura University, Mansoura, Egypt; 12 Department of Human Anatomy and Embryology, Faculty of Medicine, Mansoura University, Mansoura, Egypt; 13 Department of Basic Medical Sciences, College of Medicine, Taibah University, Madinah, Saudi Arabia

**Keywords:** folic acid, hippocampus, Iba-1, lipopolysaccharide, microglia, neuroinflammation, Notch1

## Abstract

**Introduction:**

Lipopolysaccharide (LPS) induces neuronal injury by stimulating microglia, which release pro-inflammatory markers and neurotoxic factors. Folate deficiency induces microglial activation and modulates nuclear factor-κB (NF-κB) p65 and neurogenic locus Notch homolog protein 1 (Notch1) expression in the hippocampus. This study investigated the neuroprotective effect of folic acid against LPS-induced neurotoxicity in rats, focusing on its modulation of microglial activation and the Notch1, NF-κB, and p65 signaling pathways.

**Methods:**

A total of 24 Sprague–Dawley male rats were assigned to four groups: control, folic acid, LPS, and folic acid + LPS. After sacrifice, the left cerebral hemisphere was subjected to histopathological assessment using hematoxylin and eosin (H&E) staining and immunohistochemical assessment using anti-GFAP, anti-Iba1, anti-cyclooxygenase-2 (COX-2), anti-tumor necrosis factor-*α* (TNF-α), and anti-NF-κB antibodies. The hippocampus was extracted from the right hemisphere and used to assess the gene expression of Notch1, TNF-α, interleukin-6 (IL-6), and COX-2 markers using real-time reverse transcription PCR.

**Results:**

Folic acid ameliorated LPS-induced neuronal damage in the hippocampus, suppressed microglial activation (GFAP and Iba-1), downregulated Notch1 and NF-κB p65, and improved neuroinflammatory responses (TNF-α, IL-6, and COX-2), regardless of the region.

**Conclusion:**

Folic acid exerted an equivalent neuroprotective effect in both the CA1 and CA3 regions by suppressing microglial activation and modulating the Notch1/NF-κB signaling pathway, thereby reducing neuroinflammation. These findings suggest that folic acid may serve as a potential adjuvant neuroprotective agent against inflammation-mediated neuronal injury.

## 1 Introduction

Neuroinflammation is a key trigger for neurodegenerative disorders such as Parkinson’s disease, multiple sclerosis, and Alzheimer’s disease. Lipopolysaccharide (LPS), an endotoxin, initiates inflammatory responses and induces microglial activation (Burroughs et al., 1992). Microglia are resident macrophages in the brain that act as host defenders and play a critical role in tissue repair in the central nervous system (CNS) (Perry and Gordon, 1988).

LPS induces neuronal injury through the stimulation of microglia, which release various inflammatory markers and neurotoxic factors, such as prostaglandin E2 (PGE2), nitric oxide (NO), interleukin (IL)-6, IL-1β, tumor necrosis factor-*α* (TNF-α), and cyclooxygenase (COX)-2 (Rankine et al., 2006; Ronchetti et al., 2006; Lynch, 2009; Jung et al., 2010). COX-2, an isoenzyme involved in neuroinflammation, is induced by activated inflammatory mediators and is expressed in neurons and microglia. COX-2 promotes the production of superoxide anions, which can lead to neuronal damage (Nogawa et al., 1997; Minghetti, 2007; Levy et al., 2009; Messina et al., 2009).

LPS also contributes to the activation of various genes, such as nuclear factor-κB (NF-κB), a transcription factor that normally resides in the cell cytoplasm and mediates an inflammatory response by inducing the phosphorylation and degradation of inhibitory-κB (IκB) via IκB kinase. IκB degradation prompts NF-κB to enter the nucleus and induces inflammation-associated gene transcription (Baldwin, 1996; Lee and Lee, 2002; Harari and Liao, 2010).

The NF-κB signaling pathway is pivotal for glial cell differentiation, and its activation influences developmental pathways, particularly Notch1 signaling. The interaction between developmental and inflammatory pathways has important therapeutic implications in CNS injury through the induction of neuronal regeneration (Birck et al., 2021). Notch1 signaling promotes the inflammatory reaction of motivated microglia in CNS injury. Suppression of Notch1 signaling reduces the number of activated microglial cells and the expression of inflammatory cytokines (Arumugam et al., 2006; Grandbarbe et al., 2007). Notch1 enhances the NF-κB p65 signaling pathway and triggers inflammatory mediators in LPS-stimulated microglia (Cao et al., 2011; Li et al., 2012). Blocking Notch1 partly attenuates NF-κB p65 nuclear translocation and the expression of neuroinflammatory mediators (IL-6, IL-1β, and TNF-*α*) (Cheng et al., 2019).

Folic acid is a water-soluble B-complex vitamin that protects the neural tube during development. It is essential for DNA and RNA synthesis during neural progenitor cell (NPC) proliferation and differentiation (Akchiche et al., 2012). Folate is a key factor in the regulation of acetylcholinesterase and Na+/K + ATPases, which are important for CNS functions. Folic acid deficiency can result in neurological disorders, such as depression, psychosis, dementia, and Alzheimer’s disease (Clarke, 2006). Folate deficiency also induces microglial activation in the hippocampus and modulates Notch1 and NF-κB p65 expression after ischemic injury (Cheng et al., 2019).

Suppression of microglial cell overstimulation is a target for mitigating brain injury induced by inflammatory mediators, such as LPS, to avoid progression to neurodegeneration. Thus, this study aims to elucidate whether folic acid has a neuroprotective effect against LPS-induced neurotoxicity in a rat model and whether it modulates inflammatory cytokine production by activated microglia through the Notch1 and NF-κB/p65 signaling pathways.

## 2 Materials and methods

### 2.1 Sample size

A factorial design with two factors at four and two levels has eight cells (treatment combinations) comprising four groups and two regions. A total sample size of 48 rats was needed to yield 6 rats per cell. This design provides 80% power when an F-test is used to test the grouping factor (main effect of group) at a 5% significance level, with an effect size of 0.5. This effect size was drawn from data reported in previous studies (Wen et al., 2021; Zhang et al., 2022; Valenzuela-Arzeta et al., 2023). The sample size was estimated using Power Analysis and Sample Size (PASS) software (version 2021; NCSS, LLC, Kaysville, Utah, United States, ncss.com/software/pass).

### 2.2 Experimental animals

A total of 24 male Sprague–Dawley rats aged 9–11 weeks and weighing 220–240 gm were housed under suitable temperature (24 ± 2 °C), humidity (40 ± 5%), and regular 12:12 dark/light cycles. The animals were housed at the Mansoura Medical Experimental Research Centre (MERC) with three rats per cage. All rats had free access to tap water and standard rodent chow. The animal experiments were consistent with the National Institutes of Health regulations for the care and use of laboratory animals (NIH Publication No. 8023, revised 1978) and permitted by the local Ethical Commission.

### 2.3 Chemicals used

Lipopolysaccharide powder (Sigma-Aldrich, code L2630) was administered by a single intravenous injection at a dose of 2 mg/kg body weight. For solution preparation, LPS was dissolved in sterile 0.9% physiological saline (Fu et al., 2014) and vortexed thoroughly to ensure a homogenous suspension at a concentration of 2 mg/mL. The injection volume was standardized to 1 mL/kg to ensure accuracy without causing fluid overload.

A folic acid injection vial (5 mg/mL; XGen Pharmaceuticals, DJB, Inc., United States) was injected intravenously at a dose of 4 mg/kg/day (Zhao et al., 2022), so the injection volume was 0.8 mL/kg.

### 2.4 Experimental protocol

The rats were randomized into four groups (six rats per group): the control group, the folic acid group (rats of this group were injected intravenously with folate (4 mg/kg/day) for 6 days), the LPS group (rats of this group each received a single intravenous injection with lipopolysaccharide (2 mg/kg body weight) to induce neural cytotoxicity, and rats were sacrificed after 48 h), and the folic acid + LPS group (rats were injected intravenously with folate (4 mg/kg/day) for 6 days; on the 4th day, LPS was injected 2 h after the folic acid injection).

### 2.5 Scarification of rats and specimen collection

Rats from all groups were sacrificed on the 6th day. After pentobarbital anesthesia (40 mg/kg i.p.), cervical dislocation and brain dissection were immediately performed (Magdy et al., 2022). The left hemisphere of each animal was fixed in 10% paraformaldehyde for histological and immunohistochemical analyses. For genetic studies, the hippocampus was dissected from the other hemisphere, snap-frozen, and stored at −80 °C.

### 2.6 Histological and immunohistochemical assessments

The left cerebral hemispheres were dehydrated in ascending alcohol grades, cleared in xylene, embedded in paraffin, and sectioned at 4 μm thickness. The slices were stained with hematoxylin and eosin (H&E) (H3136, 230,251, Sigma-Aldrich) (Ebrahim et al., 2023) for histological evaluation.

For the immunohistochemical study, the left cerebral hemisphere sections were immersed in hydrogen peroxide (3%) to inhibit the activity of endogenous peroxidase and then in a water bath (95 °C) with sodium citrate buffer for 30 min for antigen retrieval. The sections were left to reach room temperature and then incubated (4 °C) for 12 h with the following primary antibodies (Abcam, Cambridge, United Kingdom): anti-GFAP (ab7260, 1:500) (Dheer et al., 2018), anti-Iba1 (ab5076, 1:1000) (Bedolla et al., 2024), anti-TNF-*α* (ab220210, 1:200) (Deng et al., 2024), anti-COX-2 (ab227528, 1:200) (Qi et al., 2019), and anti-NF-κB (ab16502; 1:100) (Magdy et al., 2022). The sections were then exposed to horseradish peroxidase-conjugated secondary antibody and labelled with streptavidin-biotin (DET-HP1000, Sigma-Aldrich) for 30 min each. Finally, the reaction was visualized by adding diaminobenzidine (DAB: 3 min) and counterstained with hematoxylin.

### 2.7 mRNA quantification of Notch1, TNF-α, IL-6, and COX-2 genes by real-time reverse transcription-PCR (qRT-PCR)

The hippocampal tissue samples were homogenized, and RNA was extracted using QIAzol (Qiagen, Germany) lysis reagent. To ascertain the purity and concentration of RNA, a Thermo Scientific NanoDrop One (USA) was used to measure the yield. First-strand cDNA was created from one microgram of RNA using the Proflex (Applied Biosystems, United States) thermal cycler and the SensiFAST (Bioline, United Kingdom) cDNA synthesis kit. The primers were subjected to annealing (10 min at 25 °C), reverse transcription (15 min at 42 °C), and inactivation (5 min at 85 °C).

cDNA template amplification was performed using real-time PCR equipment (Azure Cielo 6, Azure, United States). The 20 μL reaction volume consisted of 10 μL of Bioline SYBR green PCR Master Mix (Bioline, United Kingdom), 1 μL of cDNA template, 2 μL of gene primer, and 7 μL of nuclease-free water. After a 2-min adjustment of the thermal profile (95 °C), there were 40 cycles: 5 s of denaturation (95 °C), annealing, and extension (60 °C) (but 62 °C with Notch1).

The primers were defined according to the established literature (as illustrated in Table 1). The primers’ specificity was verified using the Primer-BLAST program.^1^ Melting curve analysis was performed to confirm the specificity of the PCR products. Primer sets were obtained from Vivantis (Vivantis Technologies, Malaysia). The relative level of gene expression was calculated as ΔCt = Ct _target gene_– Ct _housekeeping gene_, and the 2^−ΔΔCT^ method was employed to estimate the fold change of gene expression (Livak and Schmittgen, 2001). The PCR products were subjected to agarose gel electrophoresis (3%) and photographed using an Azure 600 (Azure, United States) gel documentation system.

**Table 1:** Sequence of primers utilized in qRT-PCR investigation.

Gene	Sequence	Annealing temperature	Product size	RefSeq	Reference
Notch1	Forward: CACCCATGACCACTACCCAGTT Reverse: CCTCGGACCAATCAGAGATGTT	62 °C	186 bp	NM_001105721.1	Köhler et al. (2004)
TNF-α	Forward: TCTTCAAGGGACAAGGCTGC Reverse: CTTGATGGCAGAGAGGAGGC	60 °C	104 bp	NM_012675.3	Mariod and Salama (2020)
IL-6	Forward: TCCTACCCCAACTTCCAATGCTC Reverse: TTGGATGGTCTTGGTCCTTAGCC	60 °C	79 bp	NM_012589.2	Iwashita et al. (2014)
COX-2	Forward: GGAGCAACCGATGTGGAATTG Reverse: GCCGGTATCTGCCTTCATGT	60 °C	104 bp	NM_017232.4	Elhadidy et al. (2021)
GAPDH	Forward primer: TGGGAAGCTGGTCATCAAC Reverse primer: GCATCACCCCATTTGATGTT	60 °C	78 bp	NM_017008.4	Lynch (2009)

### 2.8 Morphometric study

Hippocampal sections were examined and photographed using a light microscope (Olympus^®^ CX41) equipped with a digital camera (Olympus^®^ SC100). ImageJ software (National Institute of Health, United States) was used for image analysis, where five non-overlapping fields (magnification: x400 – area: 0.069 mm^2^) from five non-consecutive hippocampal sections were quantified for an evaluation of the immunohistochemical expression of GFAP, Iba-1, TNF-*α*, COX-2, and NF-κB proteins in CA1 and CA3 regions (Table 2). To isolate the chromogen signal, images were submitted for color deconvolution using the H-DAB vector to isolate the diaminobenzidine (DAB) signal from the hematoxylin counterstain (Ruifrok and Johnston, 2001). Digital images were converted to 8-bit grayscale, and a consistent automated thresholding algorithm was applied to distinguish positive staining from background noise. The region of interest was manually defined to encompass the target tissue area, excluding artifacts. The final quantification was expressed as the area fraction, which represents the percentage of thresholded positive pixels relative to the total number of pixels within the specified area of interest. All quantifications were performed by an observer blinded to the experimental design to ensure objectivity.

**Table 2:** GFAP, NF-κB, TNFα, COX2, and Iba1 immunopositive area percentage test of interaction between groups and hippocampal regions.

Parameter	Region	Group				F-statistic	*p*-value	Partial η^2^
		Control	Folic	LPS	Folic acid + LPS			
GFAP	CA1	4.11 ± 0.66	5.03 ± 0.73	18.9 ± 1.52	6.35 ± 0.88	2.116	0.113	0.137
	CA3	4.82 ± 0.55	5.50 ± 0.87	17.7 ± 2.1	7.00 ± 0.76			
Iba1	CA1	1.67 ± 0.25	1.49 ± 0.20	10.80 ± 1.59	3.17 ± 0.30	0.522	0.669	0.038
	CA3	1.95 ± 0.18	1.64 ± 0.23	10.33 ± 1.42	3.28 ± 0.55			
TNFα	CA1	1.18 ± 0.25	0.97 ± 0.19	5.71 ± 0.96	2.51 ± 0.61	1.387	0.261	0.094
	CA3	1.17 ± 0.25	0.92 ± 0.12	6.44 ± 0.81	2.39 ± 0.78			
COX2	CA1	1.23 ± 0.26	1.17 ± 0.41	7.49 ± 0.74	2.31 ± 0.35	1.057	0.378	0.073
	CA3	1.36 ± 0.31	1.40 ± 0.45	7.01 ± 0.88	2.44 ± 0.73			
NF-κB	CA1	0.56 ± 0.32	0.91 ± 0.51	5.94 ± 1.33	2.01 ± 0.68	0.151	0.928	0.011
	CA3	0.71 ± 0.46	0.96 ± 0.58	5.96 ± 2.01	2.50 ± 0.62			

Data are expressed as mean ± standard deviation and *p*-values by two-way ANOVA. There was no statistically significant interaction effect between groups and regions on GFAP, NF-κB, TNFα, COX2, and Iba1 immunopositive area percentage.

### 2.9 Statistical analysis

A blinded statistical analysis was performed by a researcher who was only provided with letter-coded groups of datasets. The “code” is broken only once the analysis is complete. Data were analyzed using SPSS (IBM Corp., Armonk, NY), Version 25.0. Initially, the Shapiro–Wilk’s test was performed to check to assess the normality of quantitative data; the data is categorized as normally distributed if *p* > 0.05, and are reported as the mean ± standard error (SE). The presence of significant outliers was verified by analyzing the box plots. The One-Way ANOVA test was used to compare gene expression of Notch1, TNF-α, IL-6, and COX-2 between the experimental groups. To compare each pair of data, post-hoc analysis was used; the Games–Howell test was performed when the assumption of equal variances was violated, while the Tukey test was done when the assumption of equal variances was met. To evaluate the main effects of groups and hippocampal regions CA1 and CA3, as well as their potential interaction effect on GFAP, NF-κB, TNFα, COX2, and Iba1 immunopositivity, a two-way ANOVA was utilized. Post-hoc comparisons using Tukey’s HSD were performed, where significant differences were identified in the form of different letters, whereas non-significant differences were identified as similar letters. Statistical significance was considered if *p*-value was ≤ 0.050.

## 3 Result

### 3.1 Folic acid ameliorated LPS-induced neuronal damage in the Cornu Ammonis (CA1) and CA3 hippocampal regions

**Figure 1:**
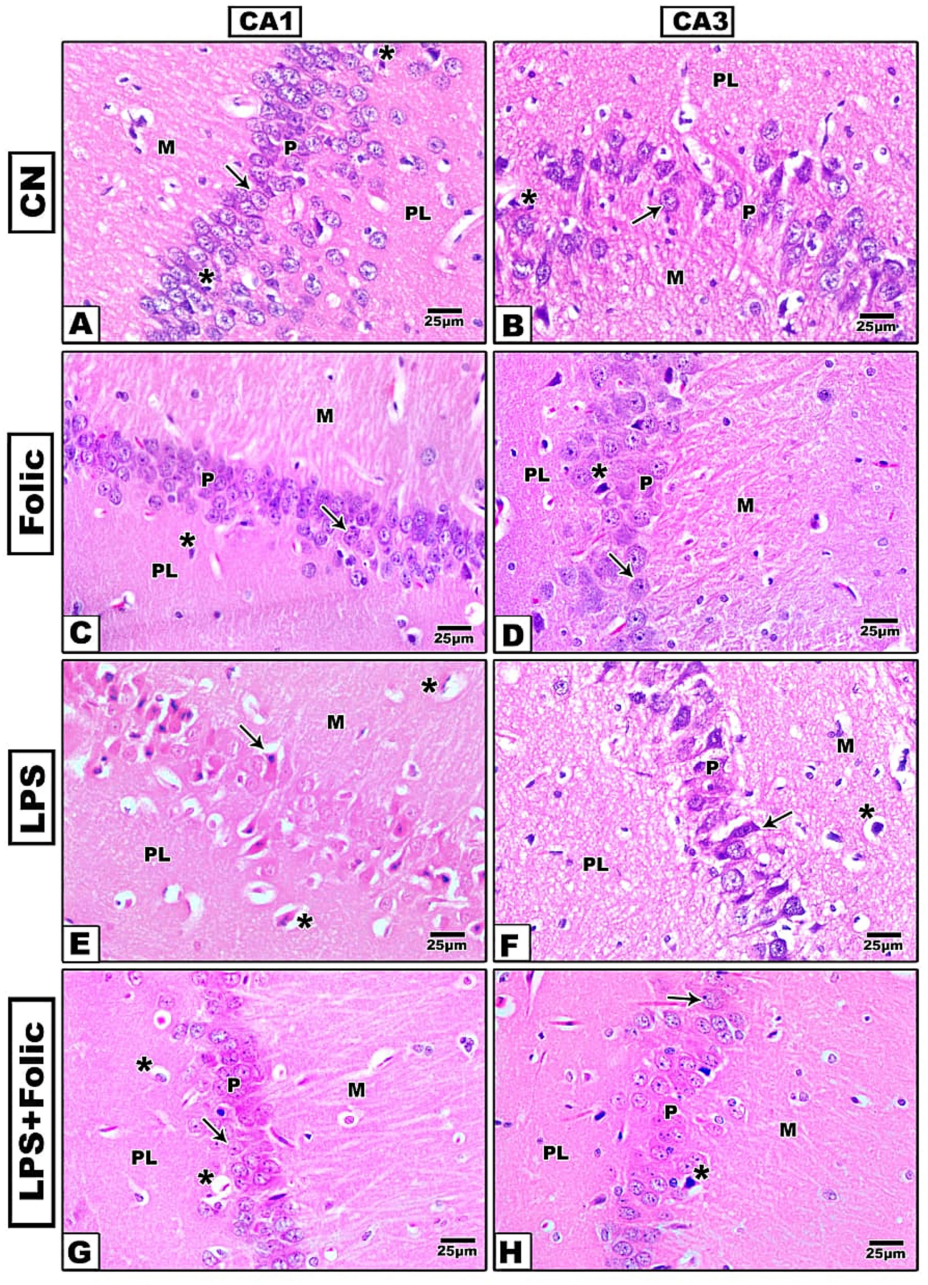
Microscopic images of H&E-stained rat hippocampus (regions CA1 and CA3) of **(A,B)** The control group and **(C,D)** The folic acid group shows small round neurons with vesicular nuclei (arrows) and sparse nuclei of neuroglial cells (*). **(E,F)** The LPS group shows degenerated neurons (arrows) and neuroglial cells with pericellular space (*). **(G,H)** The folic acid + LPS group shows a large number of normal-appearing neurons with vesicular nuclei (arrows) and few degenerated neuroglial cells with pericellular space (*). M, molecular layer; P, pyramidal layer; PL, polymorphic layer.

Histopathological changes in the CA1 and CA3 regions were detected using HE staining. In the control group (Figures 1A,B) and follicular group (Figures 1C,D), three layers were visible: molecular, pyramidal, and polymorphic. The pyramidal layer showed small, round neurons with distinct vesicular nuclei. The other two layers showed sparse neuroglial cell nuclei and small blood capillaries. The neurons in the LPS group (Figures 1E,F) were distorted, without clear cell boundaries, and the pericellular space indicated partial cell lysis. The nucleus was pyknotic (shrunken), with no nucleoli. Compared to the LPS group, cell damage was markedly improved in the folic acid + LPS group (Figures 1G,H). Specifically, when comparing CA1 and CA3 subfields, no aberrant differences were observed in the degree of neuronal preservation. While the CA1 region typically exhibits higher baseline sensitivity to the experimental insult, the folic acid + LPS group showed a comparable increase in viable pyramidal cells in both CA1 and CA3 relative to the LPS group. This parity suggests that the benefits of folic acid are distributed globally throughout the hippocampus, effectively bridging the characteristic gap in regional vulnerability.

### 3.2 Folic acid suppressed LPS-hyperactivated microglia in the CA1 and CA3 hippocampal regions

**Figure 2:**
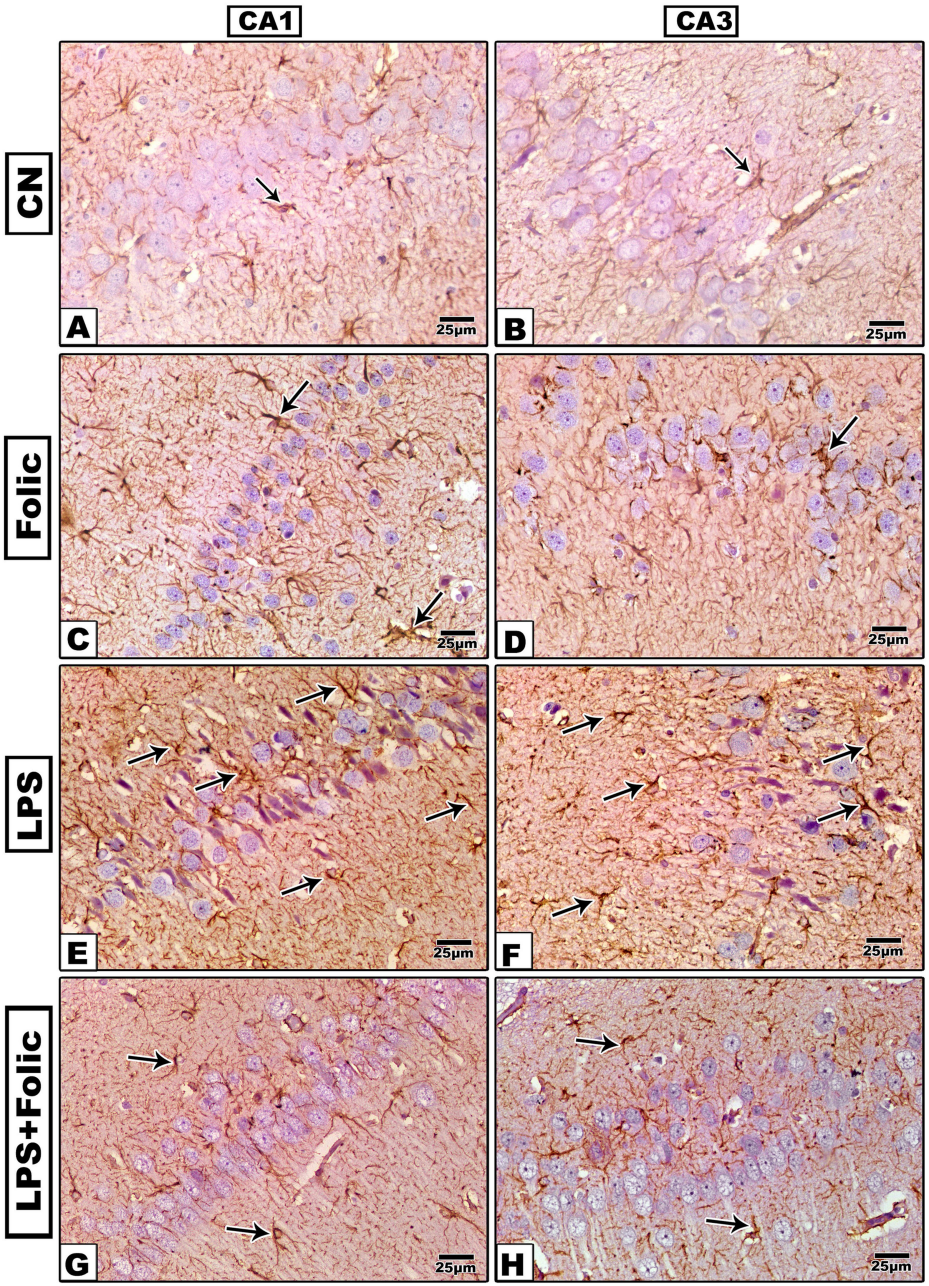
Microscopic images of the GFAP-immunostained rat hippocampus (regions CA1 and CA3) of **(A,B)** The control group and **(C,D)** The folic acid group show a mild GFAP immune reaction. **(E,F)** The LPS group shows a strong reaction. **(G,H)** The folic acid + LPS group shows a moderate reaction.

**Figure 3:**
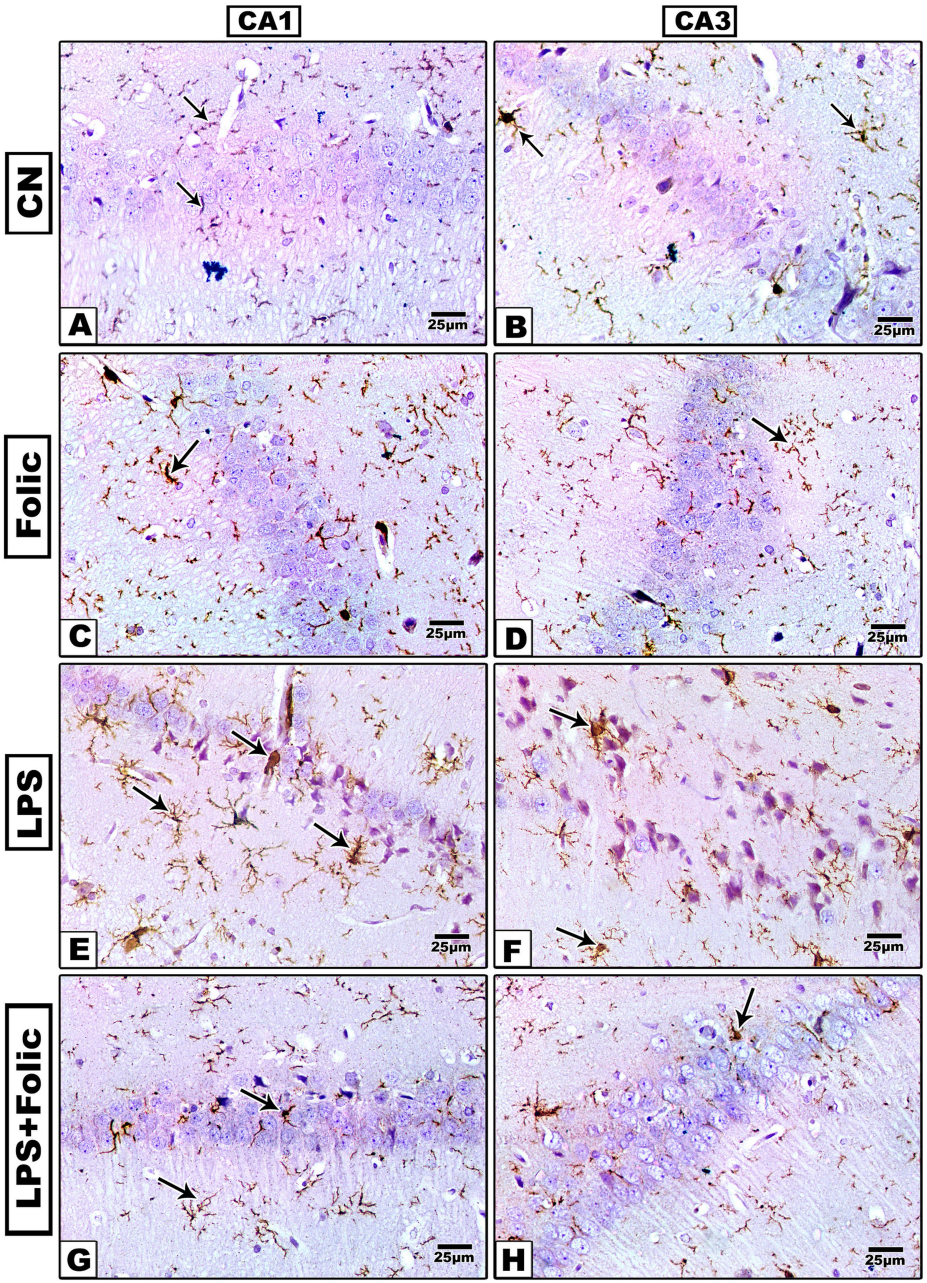
Microscopic images of the Iba-1-immunostained rat hippocampus (regions CA1 and CA3) show the amoeboid morphology of Iba-1 + microglia. **(A,B)** The control group, and **(C,D)** the folic acid group show a mild Iba-1 immune reaction. **(E,F)** The LPS group shows a marked reaction. **(G,H)** The folic acid + LPS group shows a moderate reaction.

To investigate whether LPS-induced neuronal damage was associated with the activation of microglial cells, the immunoreactivity of the microglia-specific markers GFAP and Iba-1 was examined. Two-way ANOVA was performed to test the interaction effect between groups and regions (Figures 2, 3). There was no statistically significant interaction effect between groups and regions on GFAP and Iba1 immunopositive area percentage (*p* = 0.113 and 0.669, respectively) (Table 2). On pairwise comparison of the group effect, there was a statistically significant difference between groups (*p* < 0.001); it was found that in the control group (Figures 2A,B, 3A,B) and the folic acid group (Figures 2C,D, 3C,D), there were few GFAP- and Iba-1-immunoreactive cells. The LPS group showed a significant increase in GFAP (Figures 2E,F) and Iba-1 (Figures 3E,F) immunostained area, which was significantly reduced by folic acid pretreatment (Figures 2G,H, 3G,H) regardless of the region effect (Table 3). On comparing the regions’ effects, there was no statistically significant difference in GFAP and Iba1 immunopositive area percentage between CA1 and CA3 regions, regardless of the grouping effects (*p* = 0.656 and 0.956, respectively) (Table 4).

**Table 3:** Main effect of groups on GFAP, NF-κB, TNFα, COX2, and Iba1 immunopositive area percentage.

Parameter	Groups				*F* value	*p* value	Partial η^2^
	Control	Folic	LPS	Folic acid + LPS			
GFAP	4.46 ± 0.32 A	5.26 ± 0.32 A	18.33 ± 0.32 B	6.67 ± 0.32 C	401.114	**<0.001**	0.968
Iba1	1.81 ± 0.23 A	1.57 ± 0.23 B	10.57 ± 0.23 C	3.22 ± 0.23 D	337.590	**<0.001**	0.962
TNFα	1.17 ± 0.17 A	0.94 ± 0.17 A	6.08 ± 0.17 B	2.45 ± 0.17 C	197.444	**<0.001**	0.937
COX2	1.29 ± 0.16 A	1.28 ± 0.16 A	7.25 ± 0.16 B	2.37 ± 0.16 C	310.654	**<0.001**	0.959
NF-κB	0.63 ± 0.28 A	0.93 ± 0.28 A	5.95 ± 0.28 B	2.26 ± 0.28 C	75.982	**<0.001**	0.851

Data are expressed as mean ± standard error and *p*-values by two-way ANOVA; the post-hoc test is presented as capital letters (different letters indicate statistical significance, and similar letters indicate statistical significance), with pairwise comparison using Tukey adjustment. Bold values indicate significant *p*-values (≤0.05). There was a statistically significant difference in GFAP, NF-κB, TNFα, COX2, and Iba1-immunopositive area percentage between groups, regardless of the region effect.

**Table 4:** Main effect of regions on GFAP, NF-κB, TNFα, COX2, and Iba1 immunopositive area percentage.

Parameter	Hippocampal regions		*F* value	*P* value	Partial η^2^
	CA1	CA3			
GFAP	8.61 ± 0.23	8.76 ± 0.23	0.201	0.656	0.005
Iba1	4.28 ± 0.16	4.30 ± 0.16	0.003	0.956	0.000
TNFα	2.59 ± 0.12	2.73 ± 0.12	0.663	0.420	0.016
COX2	3.05 ± 0.11	3.05 ± 0.11	0.001	0.977	0.000
NF-κB	2.36 ± 0.20	2.53 ± 0.20	0.399	0.531	0.010

Data are expressed as mean ± standard error and *p*-values by two-way ANOVA. There was no statistically significant difference in GFAP, NF-κB, TNFα, COX2, and Iba1 immunopositive area percentage between CA1 and CA3 regions, regardless of the grouping effect.

### 3.3 Folic acid improved neuroinflammatory responses in LPS-induced neuronal injury in the CA1 and CA3 hippocampal regions

**Figure 4:**
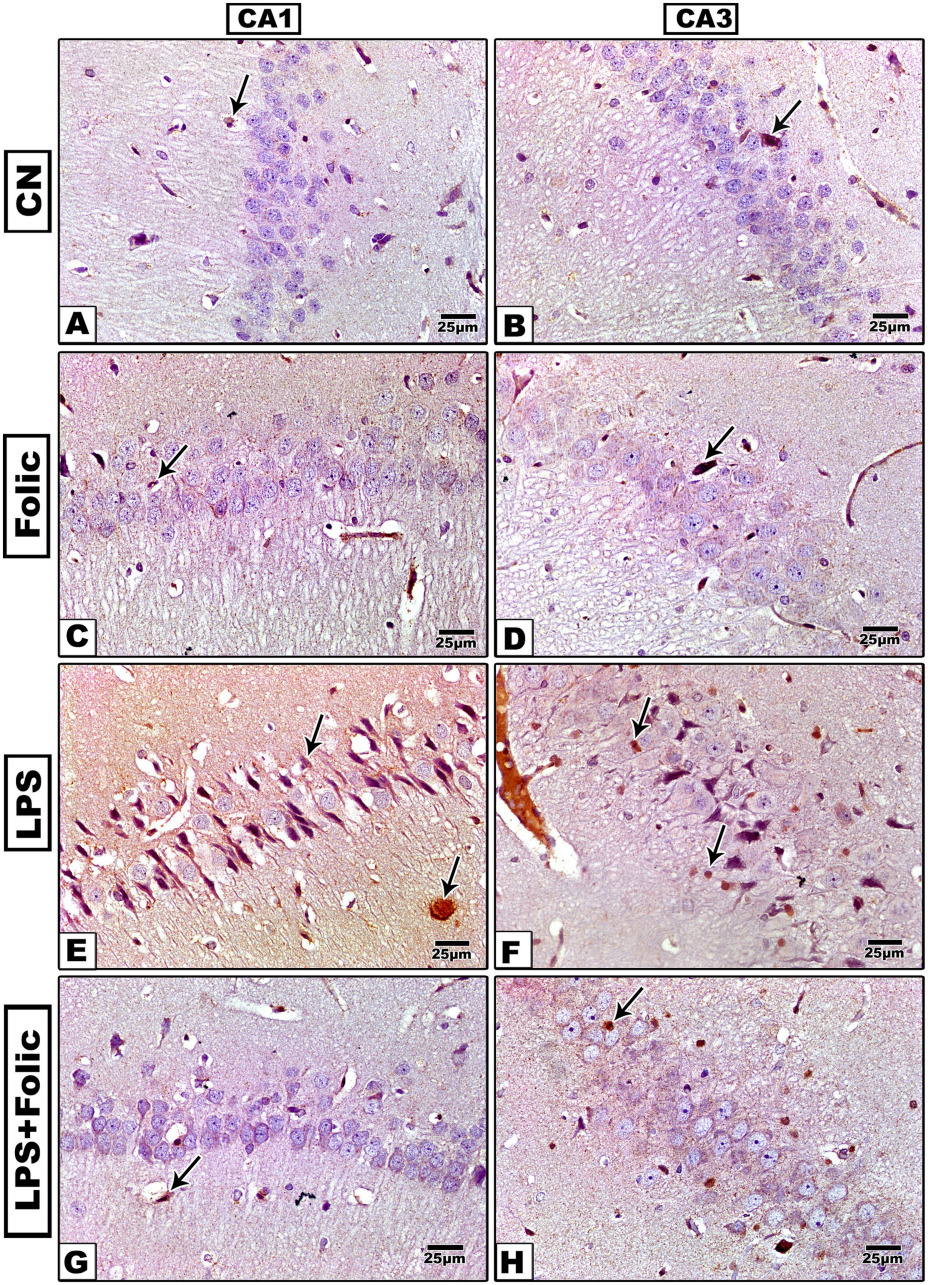
Microscopic images of the TNF-α-immunostained rat hippocampus (regions CA1 and CA3): **(A,B)** The control group and **(C,D)** the folic acid group show a weak TNF-α immune reaction. **(E,F)** The LPS group shows a very strong reaction. **(G,H)** The folic acid + LPS group shows a moderate reaction.

**Figure 5:**
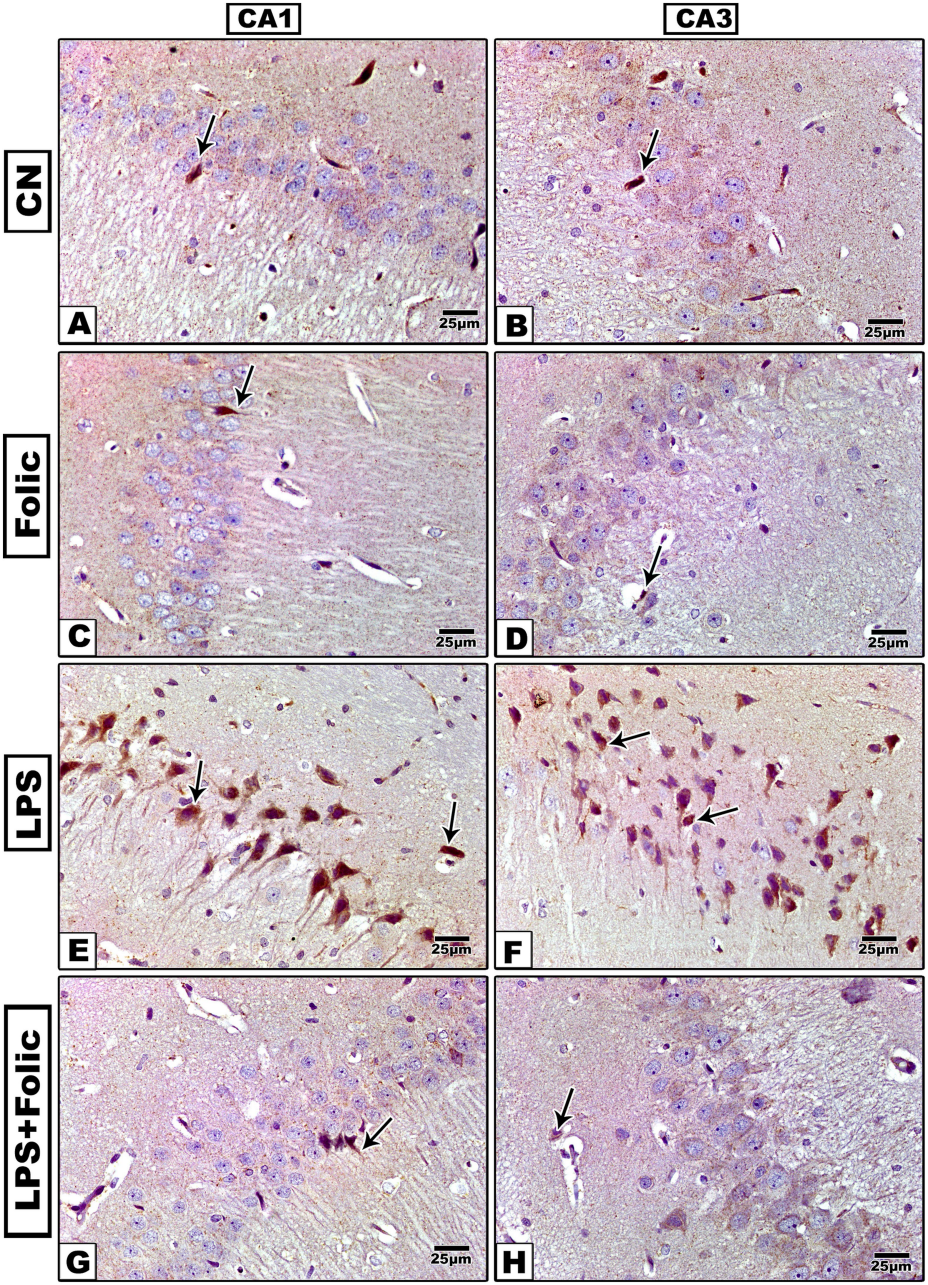
Microscopic images of the COX-2-immunostained rat hippocampus (regions CA1 and CA3) of **(A,B)** the control group and **(C,D)** the folic acid group show mild COX-2 immune reaction. **(E,F)** The LPS group shows a marked reaction. **(G,H)** The folic acid + LPS group shows a mild reaction.

To assess the neuroinflammatory response in LPS-induced injury, we examined the immunohistochemical expression of TNF-*α* and COX-2 in the CA1 and CA3 hippocampal regions. There was no statistically significant interaction effect between groups and regions on the percentage of TNF-α and COX-2 immunopositive area percentage (*p* = 0.261 and 0.379, respectively) (Table 2). On pairwise comparison of the group effect, there was a statistically significant difference between groups (*p* < 0.001); it was found that both the control group (Figures 4A,B, 5A,B) and the folic acid group (Figures 4C,D, 5C,D) showed minimal expression of TNF-α and COX-2. In the LPS group (Figures 4E,F, 5E,F), the expression of both inflammatory markers was clearly pronounced, and the immunostained area increased significantly more than that of the control group (Figures 4, 5). Pretreatment with folic acid (Figures 4G,H, 5G,H) significantly reduced the expression of neuroinflammatory markers in both hippocampal regions compared to the values in the LPS group, regardless of the region effect (Table 3). On comparing the regions’ effects, there was no statistically significant difference in TNF-α and COX-2 immunopositive area percentage between CA1 and CA3 regions, regardless of the grouping effects (*p* = 0.420 and 0.977, respectively) (Table 4).

**Figure 6:**
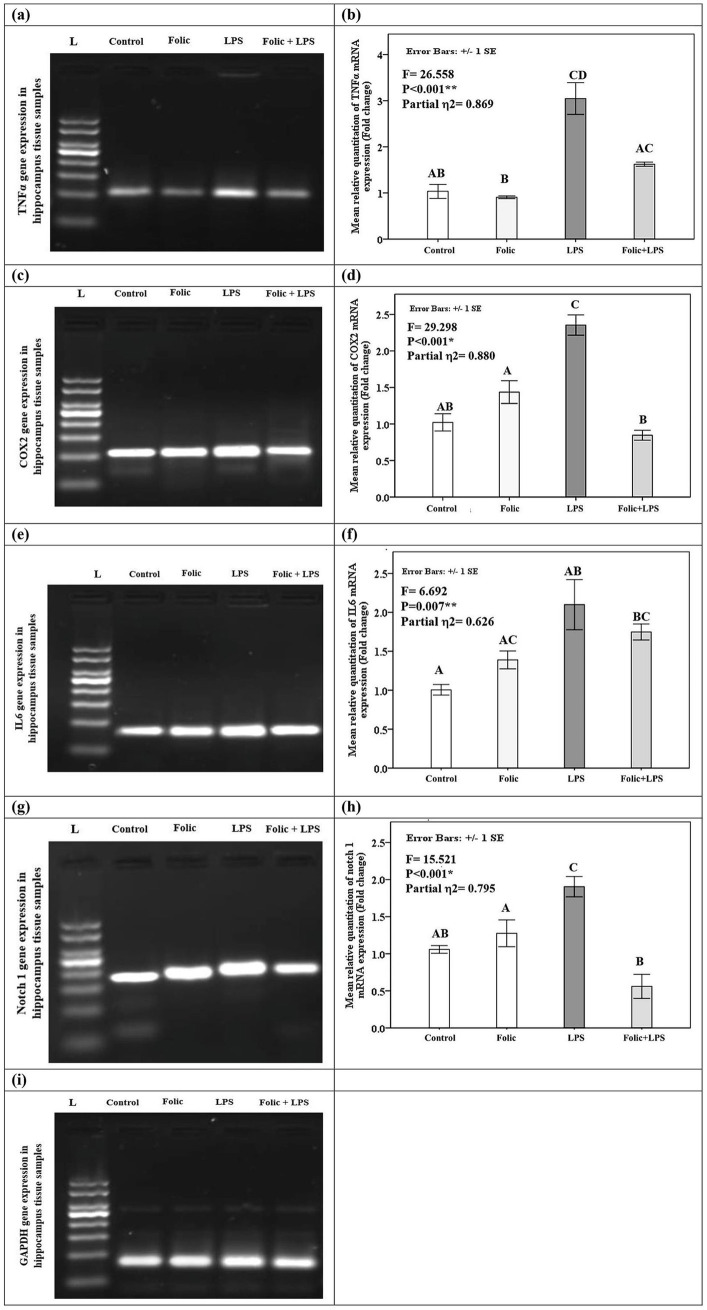
Relative quantitation of the studied genes in rat hippocampal tissue samples by real-time PCR and gel electrophoresis. **(a)** TNFα qPCR product (104 bp). **(b)** Data are expressed as mean ± standard error of TNFα gene expression fold changes. **(c)** COX2 qPCR product (104 bp). **(d)** Data are expressed as mean ± standard error of COX2 gene expression fold changes. **(e)** IL-6 qPCR product (79 bp). **(f)** Data are expressed as mean ± standard error of IL-6 gene expression fold changes. **(g)** Notch1 qPCR product (186 bp). **(h)** Data are expressed as mean ± standard error of Notch1 gene expression fold changes. **(i)** GAPDH qPCR product (78 bp). GAPDH was used as the housekeeping gene. (L) 50 bp ladder.

qPCR analysis of TNF-α (Figures 6a,b), COX-2 (Figures 6c,d), and IL-6 (Figures 6e,f) gene expression in hippocampal extracts also confirmed the significant upregulation in the LPS group that was mitigated by folic acid pretreatment (Table 5).

**Table 5:** Relative quantitation (fold change) of the studied genes in the rat hippocampus.

Parameter	Group				*F* value	*P* value	Partial η^2^
	Control (*n* = 6)	Folic (*n* = 6)	LPS (*n* = 6)	Folic acid + LPS (*n* = 6)			
TNFα	1.03 ± 0.15 AB	0.91 ± 0.03 B	3.05 ± 0.43 CD	1.62 ± 0.05 AC	26.558	<0.001**	0.869
COX2	1.02 ± 0.12 AB	1.44 ± 0.16 A	2.36 ± 0.14 C	0.85 ± 0.07 B	29.298	<0.001*	0.880
IL6	1.00 ± 0.07 A	1.39 ± 0.12 AC	2.10 ± 0.32 AB	1.75 ± 0.10 BC	6.692	0.007**	0.626
Notch1	1.06 ± 0.05 AB	1.28 ± 0.18 A	1.91 ± 0.14 C	0.56 ± 0.16 B	15.521	<0.001*	0.795

Data are expressed as mean ± standard error for gene expression and *p*-values by one-way ANOVA; the post-hoc test is represented as capital letters (different letters indicate statistical significance, similar letters indicate statistical significance), * Tukey test, ** Games–Howell test. Values in bold indicate statistically significant *p* values (≤ 0.05).

### 3.4 Folic acid decreased the expression of Notch1 and NF-κB p65 after LPS-induced injury in the CA1 and CA3 hippocampal regions

Notch1 and NF-κB p65 synergistically modulate the proinflammatory activity of microglia cells. To elucidate the underlying molecular mechanism of the LPS-induced neuroinflammatory response, Notch1 gene expression (Figures 6g,h) in hippocampal extracts was examined. The results illustrated the upregulation of Notch1 expression in the LPS-treated group (Figure 6i). Folic acid pretreatment significantly suppressed LPS-induced Notch1 expression (Table 5).

**Figure 7:**
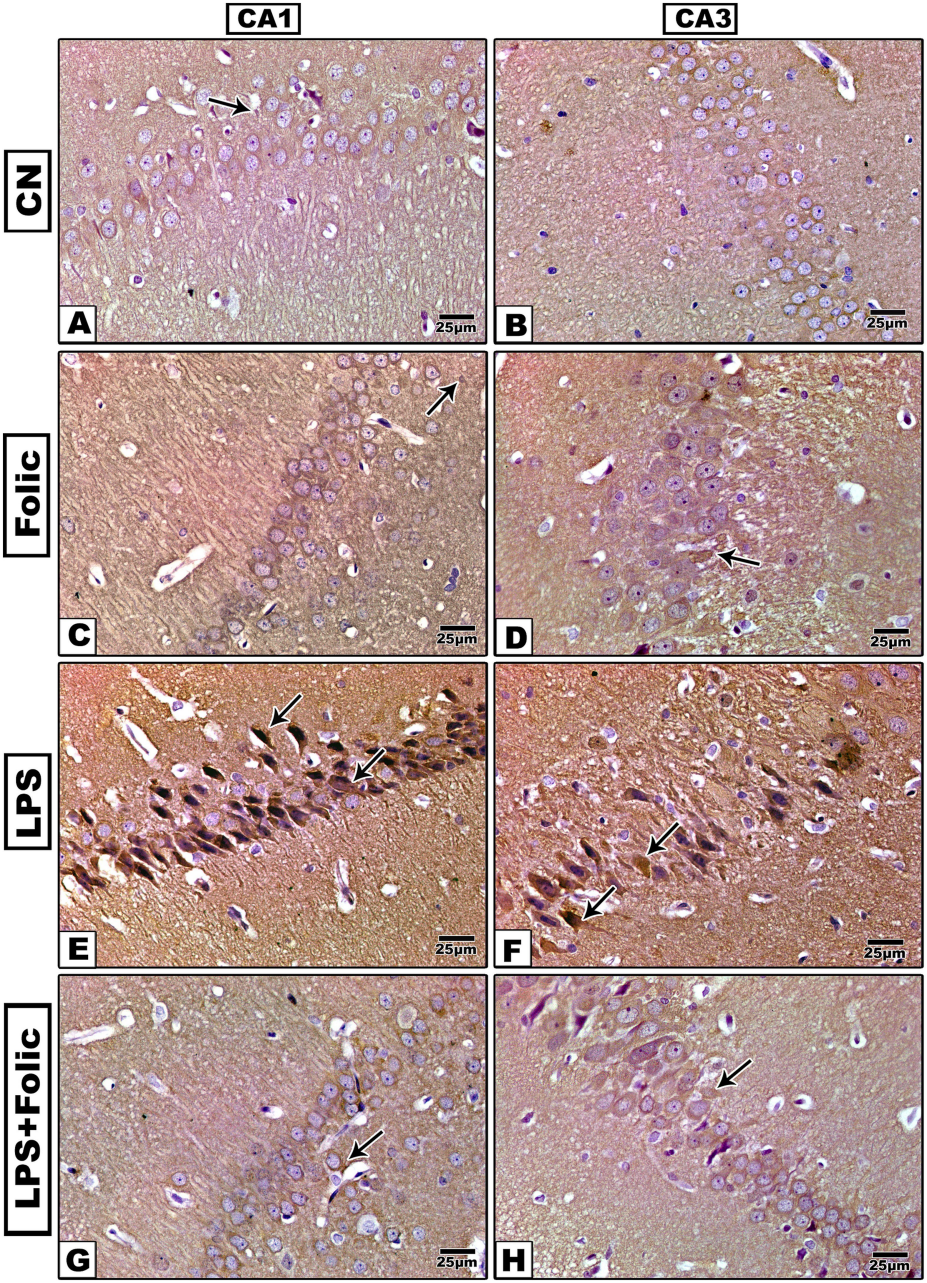
Microscopic images of the NF-κB-immunostained rat hippocampus (regions CA1 and CA3) show the characteristic nuclear immunoreaction. **(A,B)** The control group and **(C,D)** the folic acid group show a faint NF-κB immune reaction. **(E,F)** The LPS group shows a very strong reaction. **(G,H)** The folic acid + LPS group shows a mild reaction.

The immunohistochemical expression of NF-κB was examined in all experimental groups. There was no statistically significant interaction effect between groups and regions on the percentage of NF-κB immunopositive areas (*p* = 0.928) (Table 2). On pairwise comparison of the group effect, there was a statistically significant difference between groups (*p* < 0.001); it was found that the control (Figures 7A,B) and folic acid groups (Figures 7C,D) showed a weak response mainly in the cytoplasm (Figure 7). In contrast, the LPS group (Figures 7E,F) showed a strong reaction in both the cytoplasm and nucleus, whereby the immunostained area was significantly larger than that of the control group. The folic acid + LPS group showed a weak expression of NF-κB (Figures 7G,H) with a significant reduction compared to the LPS group, regardless of the region effect (Table 3). On comparing the regions’ effects, there was no statistically significant difference in NF-κB immunopositive area percentage between CA1 and CA3 regions, regardless of the grouping effect (*p* = 0.531) (Table 4).

**Figure 8:**
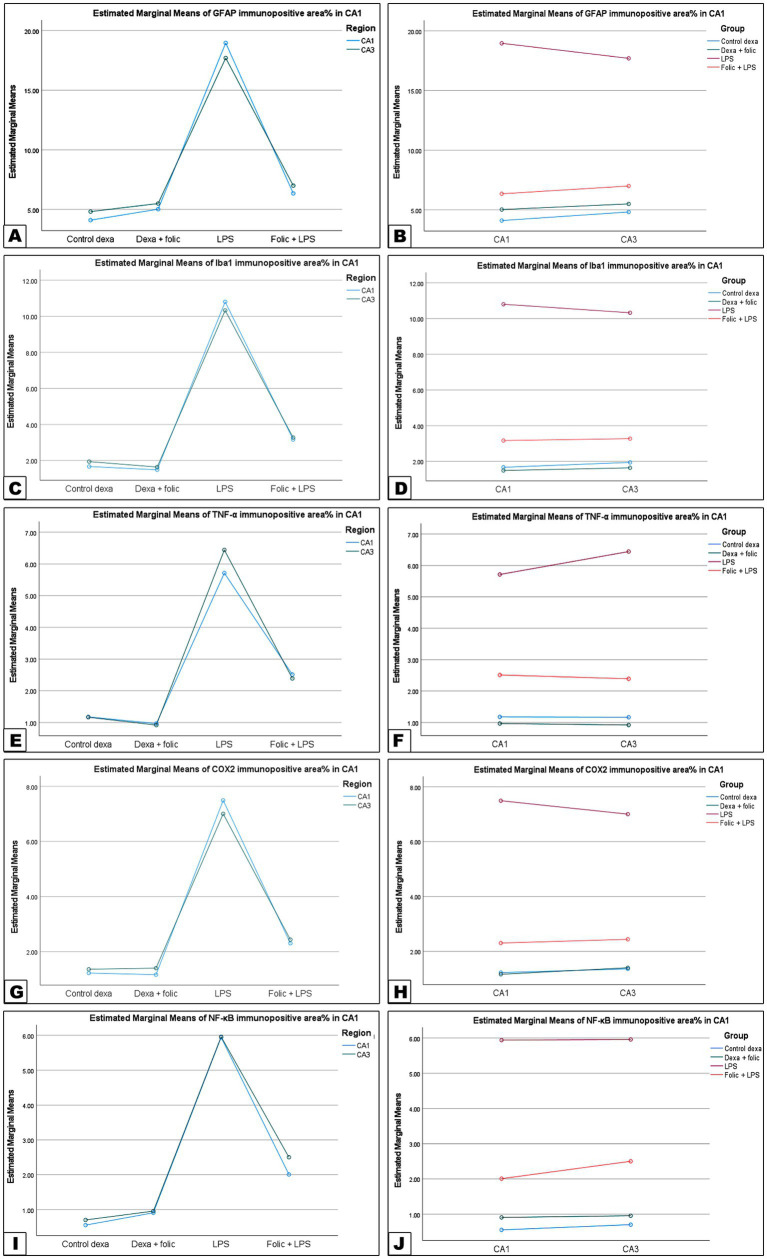
GFAP, NF-κB, TNFα, COX2, and Iba1 immunopositive area percentage test of interaction between groups and hippocampal regions. **(A)** Assessment of the regions’ effect on the percentage of GFAP-immunopositive areas. **(B)** Assessment of the grouping effect on GFAP: Percentage of immunopositive areas. **(C)** Assessment of the regions’ effect on the Iba1 percentage of immunopositive areas. **(D)** Assessment of the grouping effect on the Iba1 percentage of immunopositive areas. **(E)** Assessment of the region’s effect on the percentage of TNFα-immunopositive areas. **(F)** Assessment of the grouping effect on the percentage of TNFα-immunopositive areas. **(G)** Assessment of the effect of region on the percentage of COX2-immunopositive areas. **(H)** Assessment of the grouping effect on the percentage of COX2-immunopositive areas. **(I)** Assessment of regions’ effect on NF-κB-immunopositive area percentage. **(J)** Assessment of the grouping effect on NF-κB-immunopositive area percentage.

In summary, these results demonstrated that folic acid administration exerted a significant, and equivalent anti-inflammatory and neuroprotective effect across both the CA1 and CA3 subregions of the hippocampus in an LPS-induced model of neuroinflammation. This was evidenced by the statistically significant differences in GFAP, Iba1, TNF*α*, COX2, and NF-κB immune-positive area percentage between groups, regardless of the regions’ effects (Figure 8; Tables 2–4).

## 4 Discussion

The current study examined, for the first time, the putative prophylactic effect of folic acid against LPS-induced hippocampal neurotoxicity, focusing on microglial activation, inflammatory marker induction, and Notch1/NF-κB p65 pathway modulation in a rat model. Our results suggest that folic acid can modulate phenotypic features of microglial cells through suppression of LPS-induced neuroinflammatory markers and reduction of Notch1 and NF-κB p65 expressions.

The exact *in vivo* mechanisms by which folic acid ameliorates LPS-induced neurotoxicity remain unclear. Although many signaling cascades contribute to neuroinflammation, the Notch1 /NF-κB pathway appears as the primary driver of microglial activation. Regarding LPS injury, the Notch1 intracellular domain has been shown to synergistically interact with the NF-κB p65 subunit to induce the production of pro-inflammatory mediators such as iNOS, TNF-*α*, and IL-1β (Xia et al., 2022; Zou and Li, 2026). This specific pathway was selected because Notch1 guides a pro-inflammatory state, whereas NF-κB accomplishes the transcription of the innate immune response (Hay et al., 2025). Moreover, Notch1 was found to be predisposed to epigenetic modulation by folic acid, making this pathway a more valuable therapeutic target than other general stress-response pathways such as MAPK or Nrf2 (Zhao et al., 2022). Investigating Notch1/NF-κB aims to illuminate a more precise molecular mechanism by which folic acid maintains the microglial environment against systemic inflammatory inducers.

These data suggest that folic acid can mitigate LPS-induced neuronal damage in the CA1 and CA3 regions of the hippocampus. Histological examination demonstrated three well-organized layers in the control and follicular groups. However, neurons in the LPS group were distorted without clear cell boundaries, and the pericellular space indicated partial cell lysis. The nucleus was pyknotic and shrunken, with missing nucleoli. Moreover, the pyramidal layer contains small round neurons with distinct vesicular nuclei and the other two layers revealed sparse nuclei of neuroglial cells together with small blood capillaries. In the folic acid-pretreated group, cell damage was markedly improved, and cellular architecture was markedly restored.

The hippocampus is known to be more susceptible to neuronal injury. CA1 pyramidal neurons are more susceptible to ischemic injury than CA3 neurons (Schmidt-Kastner and Freund, 1991; Pellegrini-Giampietro et al., 1992). Nevertheless, an experimental study has shown that the CA3 hippocampal region is more susceptible to induced brain injury than the CA1 region (Mao et al., 2013). Conversely, we found that both areas were affected in our LPS-induced rat brain injury model, consistent with Cheng et al. (2019).

Folic acid pretreatment in the present study showed regression of LPS-hyperactivated microglia in the CA1 and CA3 hippocampal regions. To investigate whether LPS-induced neuronal toxicity was associated with microglial activation, immunostaining for the microglia-specific markers GFAP and Iba-1 was performed. In the control and folic acid groups, there were limited GFAP- and Iba-1-immunopositive cells. In the LPS group, a significant increase in GFAP and Iba-1 immunostained areas was demonstrated, which was significantly lessened by folic acid pretreatment in the hippocampal regions. The area percentage revealed statistically significant differences between the examined groups. Similarly, LPS elevated GFAP and Iba-1 expression in a mouse model in another study (Kang et al., 2019). Microglial cells are the principal immune cells and the first responders in the CNS, mediating neuroinflammation (Lee et al., 2014). Hyperactivity of microglia may aggravate tissue damage through the hyperproduction of inflammatory mediators, such as TNF-*α*, IL-1β, and IL-6 (Kraft and Jean Harry, 2011; Spencer et al., 2016).

Folic acid treatment also attenuated microglial activation in the hippocampus of neonatal mice in a previous study (Zhao et al., 2022). In this study, folic acid ameliorated the neuroinflammatory responses in LPS-induced neuronal injury in the CA1 and CA3 hippocampal regions, with no statistical differences between the two zones. The immunohistochemical expression of TNF-α and COX-2 in the CA1 and CA3 hippocampal regions in both the control and the folic acid groups showed minimal expression of TNF-α and COX-2. However, in the LPS group, the expression of both inflammatory markers was pronounced, and the immunostained area percentage significantly increased. Prophylactic treatment with folic acid significantly reduced the expression of neuroinflammatory markers compared to the area percentage values in the LPS group. This result is consistent with the previous result of Shin et al. (2019), which illustrated that the TNF-α antagonist counteracted LPS-induced brain injury in a rat model (Shin et al., 2019).

Interestingly, folic acid administration in this study led to equivalent neuroprotective and anti-inflammatory responses in both the CA1 and CA3 regions. The equivalent reduction in neuroinflammatory markers, specifically GFAP, Iba1, TNFα, COX2, and NF-κB across both the CA1 and CA3 subregions, suggests that FA exerts a sturdy, non- region-specific therapeutic effect. Nevertheless, the hippocampal CA1 region is considered the most vulnerable area, manifesting significant neuronal loss and neurodegeneration (Corbett et al., 2013; Gonzalez-rodriguez et al., 2021). However, folic acid provided equal protection in both zones, suggesting that it acts on a specific signaling pathway by suppressing the microglia activation, indicated by Iba1 and astrocytes, as indicated by GFAP. Folic acid effectively prevents the transition of these cells to a pro-inflammatory phenotype, thereby halting downstream cytokine production, TNF*α*, and COX2 enzymes (Szwajgier et al., 2017). The broad-spectrum efficacy of folic acid underscores its potential as a neuroprotective agent against systemic inflammation.

The immunohistochemical study results were correlated with the PCR analysis, where TNF-α, COX-2, and IL-6 gene expression in hippocampal extracts illustrated a significant increase in the LPS group, then it was mitigated significantly by folic acid pretreatment. Activated microglia give rise to inflammatory mediators, including TNF-α and IL-6, leading to neuronal damage (Dang et al., 2016; Butturini et al., 2019). Previous studies correlated LPS-induced neuroinflammation with the upregulation of inflammatory mediators such as TNF-α and IL-6 (Zhu et al., 2012). Yang et al. (2020) illustrated LPS-induced elevation in the serum levels of iNOS, IL-6, COX-2, and TNF-α and alternation in the NF-κB/MAPK p38 signaling pathways in a mouse model of neuroinflammation (Yang et al., 2020). Moreover, folate nanoparticles have been documented to alleviate LPS-induced neuroinflammation in zebrafish (Sadhukhan et al., 2022) and ameliorate LPS-induced memory deficits by alleviating oxidative stress and inflammatory reactions in rat brains (Darbandi et al., 2024).

In the current study, folic acid decreased Notch1 and NF-κB p65 expression after LPS-induced injury in the CA1 and CA3 hippocampal regions. To elucidate the underlying molecular mechanism of the LPS-induced neuroinflammatory response, Notch1 gene expression of in hippocampal extracts was investigated. The data revealed that Notch1 expression was upregulated in the LPS-treated group. However, folic acid pretreatment significantly downregulated LPS-induced Notch1 overexpression. Folate was observed to enhance cell proliferation by ameliorating Notch1 expression in rat neural stem cells (Zhang et al., 2008). Notch1 inhibition lessened NF-κB activation, attenuated inflammatory mediators, and ameliorated the neurotoxic activities of microglial cells; this could be a therapeutic target to prevent gliosis in neurodegenerative disorders (Wei et al., 2011). Notch1 also regulates microglial activation after hypoxia in rats via the NF-κB pathway (Yao et al., 2013).

It has been reported that MAPK and NF-κB signaling pathways are involved in LPS-induced neurotoxicity in brain endothelial cells (Zhou et al., 2022). In our study, the immunohistochemical expression of NF-κB was examined in all studied groups. The control and follicle groups showed weak responses, mainly in the cytoplasm. However, in the LPS group, a strong reaction both in the cytoplasm and in the nucleus was observed, whereby the immunostained area was significantly greater than that of the control group. The folic acid pretreated group demonstrated a weak NF-κB expression with a significantly reduced area percentage relative to the LPS group.

In line with our results, previous studies illustrated that folic acid deficiency stimulates microglial immune response through the Notch1/NF-κB p65 signaling pathway in the rat brain after ischemic injury (Cheng et al., 2019). Also, folic acid modulated the inflammatory response in a murine microglia cell line by regulating multiple signaling pathways, blocking NF-𝜅B activation, and inhibiting TNF-𝛼 production induced by LPS (Cianciulli et al., 2016).

Folic acid pretreatment suppressed Notch1 gene expression and attenuated NF-κB p65 protein expression. Meanwhile, LPS-hyperactivated microglia and enhanced TNF-*α* and IL-6 expression were also liable to Notch1/NF-κB p65 inhibition by folic acid. These results showed evidence that the Notch1/NF-κB p65 signaling pathway could be an underlying molecular mechanism for LPS-induced activation of microglia cells and neuroinflammation and the folic acid repair mechanism in the rat hippocampus.

Beyond the direct modulation of the Notch1/NF-κB axis, the neuroprotective effects of folic acid in our LPS model may be linked to its role in regulating acetylcholinesterase (AChE) and Na^+^/K^+^ ATPase. Recent studies have highlighted that LPS-induced microglial activation is characterized by an increase in AChE activity, which degrades the anti-inflammatory transmitter acetylcholine (Xia et al., 2022). By stabilizing AChE, folic acid likely facilitates the cholinergic anti-inflammatory response, further suppressing NF-κB-mediated cytokine release. Additionally, the preservation of Na^+^/K^+^ ATPase activity is essential; its inhibition by inflammatory injury leads to the activation of Notch1 signaling (Verkhratsky et al., 2026). Thus, folic acid acts as a multi-target regulator, maintaining enzymatic homeostasis to prevent microglia from transitioning to a pro-inflammatory phase.

Our *in vivo* results underscore the homeostatic effect of folic acid as a neuro-prophylactic mediator in LPS neurotoxicity. Moreover, our data suggest an involvement of folate in Notch1/NF-κB p65 pathway modulation, which affects microglial activation. Folic acid appears to be an important role in counteracting the inflammatory response by promoting the quiescent microglial phenotype. This could suggest the use of folic acid as a potential prophylactic supplement to protect against neuronal cytotoxicity, especially when associated with LPS.

## 5 Study limitations

Despite the favorable effects of folic acid as a neuroprophylactic agent in LPS-induced neurotoxicity, some limitations should be noted. First, in the current study, we explored the effect of folic acid on the rat hippocampus in the context of LPS neurotoxicity, however, only the immunohistochemistry and qRT-PCR techniques were illustrated. Further studies using Western blot analyses are essential to fully map out the role of folic acid in LPS cytotoxicity. Second, more advanced microscopic studies would give a more transparent image of the cellular injury/repair in LPS neurotoxicity and folate cytoprotecting. The use of only male rats is a third limitation, and future studies should investigate whether folic acid provides similar modulation in female rats across different stages of the estrous cycle.

## 6 Conclusion

In summary, these findings highlight the injurious effect of LPS on the hippocampus, which is firmly associated with microglial activation, induction of inflammatory cytokine production, and Notch1/NF-κB p65 pathway modulation. The neuroprophylactic role of folic acid is also related to the attenuation of microglial activities and suppression of TNF-α, COX-2, IL-6, Notch1, and NF-κB p65 proteins. These results elucidate the mechanisms behind the anti-inflammatory and neuroprotective effect of folic acid in LPS-induced neurotoxicity. This could guide the use of folate as an adjuvant neuroprotector to avoid the drawbacks of neurotoxic agents, especially LPS.

### 6.1 Prospective study

Further clinical trials would be valuable to confirm our hypothesis that folic acid is an effective neuroprotective agent against LPS and various brain injuries.

## Data Availability

The raw data supporting the conclusions of this article will be made available by the authors, without undue reservation.

## Footnotes

1
https://www.ncbi.nlm.nih.gov/tools/primer-blast/
